# Supplementary material for: Are carbon emissions trading and green financial instruments synergistic? -Comprehensive quantitative research based on content analysis
Source: PLoS One. 2024 Mar 7;19(3):e0298601. doi: 10.1371/journal.pone.0298601 (PMC10919601; doi:10.1371/journal.pone.0298601)
Supplement: S1 Data — (ZIP) [file pone.0298601.s001.zip › Data/地级市Z_F工作报告.zip!/╣≤╓▌/┴∙┼╠╦«╩╨/└┤╫╘╣1⁄2╓┌║┼í╛┬φ┐╦╩2╛▌═°í┐_2016.docx]

各位代表：

　　现在，我代表市人民政府向大会作工作报告，请予审议，并请市政协委员和其他列席人员提出意见。

　　一、“十二五”时期工作回顾

　　“十二五”是我市发展极不平凡的五年。在市委的坚强领导下，在市人大、市政协的监督和支持下，我们深入学习贯彻习近平总书记系列重要讲话精神，认真落实党中央、国务院和省委、省政府各项决策部署，协调推进“四个全面”战略布局，坚持主基调主战略，牢牢守住发展、生态、安全三条底线，统筹做好稳增长、促改革、调结构、惠民生、防风险各项工作，实现了“十二五”圆满收官，进入后发赶超、同步小康的重要阶段。

　　——这是综合实力显著增强的五年。坚持以加快发展为第一要务，综合实力持续攀升。全市生产总值达到1201.08亿元，年均增长14.9%；固定资产投资累计完成5280.03亿元，年均增长42.6%；公共财政预算收入达到130.26亿元，年均增长21.45%；社会消费品零售总额达到292.72亿元，年均增长17.4%；金融机构存款、贷款余额分别达到976.8亿元和779.74亿元，年均增长14.4%、16.7%；城镇、农村居民人均可支配收入分别达到23327元、7522元，年均增长11.5%、15.6%；全面建成小康社会指数提高到80.8%，年均提高3.9个百分点；盘县进入全国百强县、西部十强县行列，钟山区、水城县、六枝特区进入全省经济强县行列。

　　——这是转型发展提速增效的五年。大力优化产业结构，三次产业结构从2010年的6∶60.6∶33.4调整为9.5∶51.1∶39.4。围绕“百姓富、生态美”推动一产转型，启动实施农业特色产业“3155工程”并完成320.75万亩，粮经比从2010年的70∶30调整为36∶64；一产增加值增速连续两年排名全省第一，助农增收综合考核连续五年排名全省第一。成功获批第七批国家农业科技园区；创建省级现代高效农业示范园区26个，其中引领型园区8个。围绕“结构好、效益高”推动二产升级，循环经济示范城市获国家批复，黔桂天能等一批循环经济项目建成投产；煤矿正常生产矿井机械化程度达到69%，煤炭资源就地转化率达到70%，煤层气开发利用率达到40%，工业固体废弃物综合利用率达到68%；建成11个产业园区，其中省级产业园区5个，产业园区产值年均增长24%；大数据电子信息、大健康医药、新型建筑建材等产业发展风生水起。围绕“市场活、可持续”推动三产优化，以建设山地健康旅游目的地城市为目标，精心精准打造“中国凉都”山地旅游升级版，成功举办第八届贵州旅游产业发展大会，培育冬季滑雪、温泉度假、户外运动、农旅文融合发展等旅游新业态，初步彰显了我市山地特色旅游的独特魅力。全市接待游客和旅游总收入年均分别增长35.3%、47.4%，山地旅游成为新的经济增长点。引进银行、保险和证券机构11家。钟山区成功进入全省10个现代服务业发展示范区。

　　——这是基础设施突飞猛进的五年。交通建设取得历史性突破，安六城际铁路、沪昆客专境内段建设加快；高速公路通车里程达到320公里，实现县县通高速、乡乡通油路、村村通公路；月照机场建成通航，航班通达北京、上海、广州等11个城市。水利建设取得重大进展，建成双桥、鱼洞坝、旧院等5座中型水库和8座小型水库，水库库容达到3亿立方米；开发利用地下水机井400口，累计解决117.72万农村人口饮水安全问题。电网、信息等建设取得新成绩，建成输电线路5334公里、35千伏及以上变电站45座，主网实现双环网供电。推进“宽带六盘水”、农村地面数字电视建设，全面实现“乡乡通宽带”“村村通电话”，行政村通宽带覆盖比例达到100%。市中心城区实现4G全覆盖。

　　——这是城乡面貌焕然一新的五年。全面推进山地特色新型城镇化，全市常住人口城镇化率达到47.5%。完成第四轮城市总体规划修编。加快推进钟山、水城、红桥和市中心城区、六枝、盘县两个“三地同城化”。成功进入全国首批地下综合管廊建设试点城市和首批国家智慧城市试点。建成机场高速、内环快线等城市道路，建成贵州三线建设博物馆、凉都体育中心、六个城市湿地公园等公共服务设施，开工建设水城河综合治理工程等重大项目，实施市中心城区249个城市山体保护规划和景观提升，城市人居环境显著改善。大力开展“四创”活动，我市获“全国文明城市”提名，盘县获“国家卫生县城”称号和“全国县级文明城市”提名，“多彩贵州文明行动”考评连续两年排名全省第一。启动建设34个特色小城镇，创建省级示范小城镇5个、省级样板镇1个；六枝郎岱等9个镇被列为全国重点镇，盘县妥乐古银杏景区保护与治理项目获中国人居环境范例奖。“四在农家·美丽乡村”六个小康行动计划完成投资82.5亿元，建成美丽乡村省级示范点21个，农村面貌发生显著变化。

　　——这是生态文明建设卓有成效的五年。坚持把生态做成产业、把产业做成生态，按照“四个最大化”要求，大力实施“绿色贵州”建设六盘水三年行动计划，累计完成营造林215.23万亩，治理石漠化299平方公里、水土流失1074.91平方公里；森林覆盖率达到48.5%，比2010年提高11.5个百分点。成功获得“三乡五园”八张国家级重要生态名片。钟山区被列为全省主体功能区6个重点开发区之一。发出“美丽凉都拒绝污染”强音，狠抓环境污染治理，建成14座污水处理厂等环保设施，城镇污水处理率和城乡生活垃圾无害化处理率分别达到91.6%和88%。淘汰落后产能452.75万吨，单位生产总值能耗下降19.78%，完成“十二五”节能减排指标任务。深入实施重点流域河长制，三岔河流域内考核连续两年排名全省第一。启动空气质量AQI监测并定期发布监测数据，全市空气质量API优良率达到99.4%。开展森林保护“六个严禁”和环境保护“六个一律”利剑行动，严肃查处一批生态环保违法案件。

　　——这是改革开放全面深化的五年。启动3批189个市级重点改革专题，完成改革任务128项。创造性地开展“三变”改革，得到中央领导和省委、省政府充分肯定并在全省推广，六枝特区、盘县、水城县进入全国农村“两权”抵押贷款试点。资源配置、国有企业、财税金融、社会事业、司法体制等改革不断深化。进一步扩大对外开放，积极融入国家“一带一路”和长江经济带规划，六盘水海关获国家批复设立，成功举办生态文明贵阳国际论坛六盘水分论坛、第三届川滇黔十市州合作与发展峰会。荣获2015年“中国十佳投资环境城市”，引进500强企业23家，招商引资实际到位资金累计完成2285亿元；实际利用外资总额累计完成10.02亿美元，年均增长62.61%。民营经济完成增加值661亿元，占全市GDP比重达到55%。国家科技“863”计划项目实现零的突破，高新技术产业产值累计完成263亿元，综合科技进步水平指数提高到41.4%，人才总量达到27.4万人。

　　——这是发展成果更加惠民的五年。每年启动实施一批民生工程和民生实事，累计完成投资307.5亿元。大力实施精准脱贫，实现盘县国家扶贫开发重点县和61个贫困乡镇“减贫摘帽”，实施241个贫困村“整村推进”，累计减少贫困人口55.99万人，累计实施易地扶贫搬迁8752户、34712人。加快创建国家级创业型城市，城镇新增就业累计达到34.24万人，城镇登记失业率控制在4.2%以内。大力实施“4+2”等教育工程，从2013年起每年压缩行政经费5%用于实施教育“9+3”计划，启动基本普及15年教育，开工建设高中教育城、以朵职教城和六盘水师范学院二期、六盘水职业技术学院二期等重点工程。创建5家三级医院，开工建设凉都医院；新型农村合作医疗参合率达到99.12%，城乡居民大病保险实现全覆盖；全面实施单独二孩政策，人口计生实现“三降一升”目标。高标准举办消夏文化节系列活动，凉都夏季国际马拉松赛获“金牌赛事”称号；新增全国重点文物保护单位2个，成功申报2项国家级非遗名录；群众体育和竞技体育取得新进步。城镇和农村低保标准分别年均增长16.67%和22.48%。实施城镇保障性住房43150套，改造农村危房159781户。加强农村留守儿童、困境儿童和留守老人精准关爱救助保障。安全生产事故起数和死亡人数连续五年实现“双降”。全市群众安全感满意度达到94.43%，较2010年提高14.68个百分点。连续两年获“全国双拥模范城”称号。各项事业取得新成绩。

　　——这是自身建设持续加强的五年。自觉在市委的领导下开展工作，重大决策向市人大常委会报告、向市政协通报、向社会公开征求意见，自觉接受各方监督。深入开展党的群众路线教育实践活动和“三严三实”专题教育，坚决落实中央、省、市作风建设各项规定。制定和公布市级35个行政机关权力清单和责任清单。取消行政审批事项144项，下放审批权限事项165项，办理时限压缩率达到65%。全面推进政务公开和“阳光晒权”活动，率先在全省建成公共资源交易中心和市、县网上政务办事大厅站点。“六五”普法通过省级验收。认真落实党风廉政建设主体责任，加强行政监察和审计监督，全面推进民生监督工作，勤政廉政建设取得新成绩。荣获2015年“中国十佳诚信政府”称号。

　　刚刚过去的2015年，我们坚持把保总量、保速度、保进位作为首要任务，强化运行调度和精准服务，经济社会发展呈现总体平稳、稳中有进、进中有忧、忧中有喜的态势。全市生产总值同比增长12.1%，2000万元以上工业增加值增长10%，固定资产投资增长25.9%，公共财政预算收入增长1.18%，城镇、农村居民人均可支配收入分别增长10.2%和10.8%，社会消费品零售总额增长12%，金融机构存款、贷款余额分别增长19.5%和11.2%。“十件民生实事”完成投资50.6亿元。

　　各位代表！五年的成就令人振奋，积累的经验弥足珍贵。我们深深体会到，推动六盘水同步全面建成小康社会，必须坚持上接天线与下接地气相结合，既要以中央和省委、省政府的重要思想、重要精神为指引，又要了解群众心声、回应群众期待；必须坚持把握大势与发挥优势相结合，与时俱进学习贯彻中央和省委、省政府重大决策部署，立足本地实际，创造性地抓好贯彻落实；必须坚持盘活存量与培育增量相结合，既要立足煤、做足煤的文章，加快传统产业转型升级，又要不唯煤、大力发展新兴产业，培育新的经济增长点；必须坚持深化改革与扩大开放相结合，以改革促开放、以开放倒逼改革，不断释放改革红利，激发内生动力，提升发展活力；必须坚持绿水青山与金山银山相结合，坚持生态产业化、产业生态化，既要绿水青山又要金山银山，把生态之美化作百姓之富；必须坚持加快发展与改善民生相结合，突出抓好脱贫攻坚第一民生工程，统筹解决好教育、医疗、就业、社保、养老、住房等民生问题，让人民群众共享改革发展成果。

　　过去五年的成绩，是省委、省政府和市委正确领导的结果，是市人大、市政协监督支持的结果，是全市各族干部群众群策群力、艰苦奋斗的结果。我代表市人民政府，向全市各族人民、各级干部表示崇高的敬意和衷心的感谢！向人大代表、政协委员，向各民主党派、工商联、无党派人士、人民团体，向驻市人民解放军、武警消防官兵和人民警察，向所有关心和支持我市改革发展的各界人士，表示衷心的感谢！

　　在肯定成绩的同时，我们必须清醒地认识到存在的困难和问题。主要是：贫困人口多、贫困程度深的状况没有改变，城乡差距大，脱贫攻坚任务艰巨；经济下行压力趋紧，传统工业企业生产经营困难，财政收支矛盾突出，调结构转方式任务艰巨；上学难、看病难、养老难、就业难等问题仍不同程度存在，安全生产保障能力不足，民生保障兜底任务艰巨；少数干部慢作为、不作为、乱作为的现象依然存在，违规违纪现象时有发生，个别领域腐败易发多发，政府职能转变任务艰巨。我们将正视问题，采取更加有效的措施，切实加以解决。

　　二、“十三五”时期的主要目标任务

　　“十三五”时期我市经济社会发展的总体要求是：高举中国特色社会主义伟大旗帜，全面贯彻落实党的十八大和十八届三中、四中、五中全会及省委十一届六次全会、市委六届九次全会精神，深入贯彻习近平总书记系列重要讲话精神，统筹推进“五位一体”总体布局，协调推进“四个全面”战略布局，贯彻创新、协调、绿色、开放、共享五大发展理念，坚持主基调主战略，守住发展、生态、安全三条底线，突出抓好大扶贫、大数据、大健康三大战略行动，突出抓好循环经济、农村改革、山地旅游三件大事，突出走好山地特色产业生态路、山地特色现代农业路、山地特色健康旅游路、山地特色城乡一体路四条新路，奋力谱写守底线、走新路、奔小康新篇章，确保与全国全省同步全面建成小康社会。

　　“十三五”时期我市经济社会发展的主要预期目标是：地区生产总值年均增长12%以上；现行标准下农村贫困人口全部脱贫，贫困县乡全部摘帽，贫困村全部退出；2000万元以上工业增加值年均增长10%以上，固定资产投资年均增长20%左右，社会消费品零售总额年均增长12%；公共财政预算收入年均增长6%，城镇、农村居民人均可支配收入年均分别增长11%和12%左右，城镇新增就业35万人以上、城镇登记失业率控制在4.2%以内；城镇化率提高到55%以上；新兴产业占生产总值的比重提高到20%、服务业比重提高到45%左右；平均受教育年限、平均预期寿命达到全面小康目标；森林覆盖率达到60%以上，完成省下达的节能减排指标任务。通过五年努力，经济社会发展实现“一方阵五前列”。

　　守底线、走新路、奔小康是“十三五”时期的总纲，三大战略行动、三件大事、四条新路是总路径。我们将坚定总纲、总路径不动摇，培植后发优势，奋力后发赶超，努力走出一条不同于东部、有别于西部其他地区的发展新路。今后五年，我们将重点抓好以下工作。

　　（一）坚持以脱贫攻坚为统揽，同步全面建成小康社会。深入贯彻科学治贫、精准扶贫、有效脱贫基本方略，把脱贫攻坚作为“十三五”时期的头等大事和第一民生工程来抓，大力实施大扶贫战略行动，举全市之力、集全市之智，坚决打赢脱贫这场输不起的攻坚战。全力落实脱贫任务。深入实施“33669”脱贫攻坚行动计划，明确时间表、制定作战图、打好歼灭战，决战三年、摆脱贫困，确保到2020年与全国全省同步全面建成小康社会。精准落实脱贫措施。坚持脱贫攻坚“八个精准”，认真落实“1+10+6”文件。大力实施“五个一批”，加快贫困地区特色优势产业发展，实现村村有特色主导产品、乡乡有产业扶贫基地、县县有高标准农业示范园区和大型农产品加工龙头企业；狠抓易地扶贫搬迁，实施易地扶贫搬迁建档立卡贫困户10.7万人；积极争取中央重点生态功能区转移支付，贫困地区25度以上坡耕地和15度至25度重要水源地坡耕地实现退耕还林还草；完成农村幼儿园、寄宿制学校标准化建设，全部免除（补助）农村贫困家庭学生就读高中、大学的学杂费，不让贫困家庭的孩子输在起跑线上；完成贫困地区县乡村医疗卫生服务网络标准化建设，减少农民群众因病致贫返贫；实现“两线合一、减量提标”，织牢民生网底。严格落实脱贫责任。强化脱贫攻坚六大责任，立下军令状、用好指挥棒，层层传导压力抓落实。发挥大连对口帮扶优势，深化“千企帮村、万户结对”活动，统筹好专项扶贫、行业扶贫、社会扶贫等多方力量，积极构建大扶贫格局。

　　（二）坚持以循环经济为旗帜，大力推进工业转型升级。围绕建成国家循环经济示范城市目标，高举循环经济旗帜，加快建立绿色循环低碳的现代工业体系。抓好全域循环化发展。坚持把循环经济贯穿到生产、流通、消费的全过程，贯穿到一产、二产、三产的各方面，全面推进产业循环、城乡循环、社会循环，加快建设一批循环经济示范县、示范乡镇、示范园区、示范行业和示范企业。抓好工业循环化改造。坚持立足煤、做足煤、不唯煤，加快传统产业生态化、循环化、绿色化改造，推动煤电钢等传统产业向精深加工转型升级，延长产业链、拓宽产业幅、提高附加值。大力推进煤炭清洁生产，提高煤炭生产安全水平、集约化水平。加大煤层气、页岩气等新能源开发利用力度，推进煤炭地下气化项目建设。加快局域电网建设，推进电能就地转化利用。到2020年，煤炭资源就地转化率达到80%以上，建设中国南方现代煤电化一体化基地、西南地区重要的能源基地、贵州非常规天然气示范抽采基地。抓好资源循环化利用。大力推进煤矸石、矿渣、建筑废料、粉煤灰等大宗固体废弃物综合利用，加强生活垃圾分类回收和再生资源回收，实现资源“吃干榨尽”“变废为宝”。

　　（三）坚持以山地特色为主导，全力培植后发赶超优势。做好山地特色农业文章。围绕打造喀斯特山区特色农业示范区目标，按照强龙头、创品牌、带农户的思路，以国家农业科技园区建设为载体，以“3155工程”为抓手，加强品种品质品牌联动和市场培育，提高经济作物占种植业、畜牧业占农业总产值的比重。到2020年，特色农业产值达到100亿元以上，粮经比调整到20∶80左右。培育壮大龙头企业、农民专业合作组织等农业经营主体，培养新型职业农民。大力发展农产品精深加工，延伸农业产业链条，实现农业“接二连三”。做好山地特色旅游文章。围绕建设山地健康旅游目的地城市，以大交通带动大旅游、以大数据助推大旅游，促进农旅、文旅、工旅等深度融合，加快发展避暑纳凉、休闲度假、冬季滑雪、户外运动等山地旅游新业态，不断丰富“中国凉都”品牌内涵。加快推进“智慧旅游”建设，重点打造一批具有唯一性和竞争力的重点景区景点，完善配套设施和功能，提升服务管理水平。到2020年，接待游客2000万人次以上，旅游总收入达到260亿元左右。做好山地特色城镇文章。遵循城市发展规律，统筹空间规模产业三大结构、规划建设管理三大环节、改革科技文化三大动力、生产生活生态三大布局、政府社会市民三大主体，全面提高城市工作水平。加强城市设计，优化城市空间布局，加快推进两个“三地同城化”融合发展。加强城镇地上地下基础设施建设，积极争取海绵城市试点，建成城市地下综合管廊等项目，加快完善城镇道路网络，推进市中心城区城市轨道交通立项工作。加快智慧城市建设，提升城市管理智能化水平。打造一批特色小城镇，推进城镇棚户区和工矿企业棚户区改造，培育一批区域性中心村和示范村，促进城乡统筹、协调发展。

　　（四）坚持以大数据、大健康行动为抓手，不断发展壮大新兴产业。把大力发展大数据产业、大健康产业“姊妹篇”作为我市弯道取直、后发赶超的战略引擎来抓。大力实施大数据战略行动。加快引进落地一批大数据优强企业，建成北斗大数据、中国网络菜市场等一批产业园，推进“云上贵州·安全云”运用中心等大数据项目建设。大力发展农村电子商务、跨境电子商务，打通“黔货出山·凉都珍好”和“网货下乡”双向通道。全力推进大数据招商引智工作。到2020年，大数据电子信息产业产值达到180亿元左右。大力实施大健康战略行动。围绕“医、养、健、管”，推进大健康六大产业体系建设。突出“医养”结合，加快发展大健康医药医疗产业，积极发展中药材及民族药材、中成药及民族药品，打造大健康医药和大健康医疗品牌。突出“健养”结合，加快发展绿色健康、养身养老、户外运动等产业。到2020年，大健康产业产值达到200亿元左右。大力发展其他新兴产业。加快发展具有产业基础和市场前景的装备制造业。大力发展天然饮用水产业，力争到2020年产值达到100亿元左右。建设一批新型建材产业基地。加快发展现代金融、仓储物流等服务业。

　　（五）坚持以互联互通为目标，加快完善基础设施网络。加快完善综合交通运输网络。建成沪昆客专境内段、安六城际铁路，加快推进毕水兴城际铁路水城至盘县段、六盘水经威宁至昭通城际铁路立项工作。建成盘兴、六威高速公路，加快推进纳兴高速、宜六高速赫章至六盘水段建设，形成“三横两纵一环线”的主骨架。加快国省道、县乡道提等改造步伐，实现90%国道、30%省道提等为二级以上公路，力争全市乡镇实现二级路全覆盖，100%行政村通油路、通客车。培育和加密月照机场航线，到2020年进出港旅客突破50万人次；开工建设盘县机场并力争“十三五”建成。推进北盘江航运建设。加快完善水利工程网络。围绕县县有中型水库、乡乡有稳定供水水源目标，规划建设一批保障工业化、城镇化、农业现代化用水的骨干水源工程。大力推进盘县英武和水城县彭家桥两座大型水库前期工作，力争“十三五”开工建设。加强供水管网工程和民生水利保障工程建设维护管理。实施农田水利配套工程。加快完善信息基础设施网络。深入实施“宽带六盘水”工程，加快推进“三网融合”。到2020年，力争实现自然村“村村通宽带”。推进“无线城市”建设，加快手机免费无线上网工程建设，实现4G、无线局域网城乡全覆盖。加快完善能源基础设施网络。建成一批高效率的输变电站及大容量输电走廊。规划建设煤层气、页岩气、焦炉煤气输送管网工程，建设一批LNG工厂和加气站。建成中缅天然气管道六盘水支线工程。

　　（六）坚持以生态文明为引领，推动生产生活生态绿色化。在“建”上下功夫。大力实施退耕还林、荒山造林、石漠化治理等生态建设工程，确保森林覆盖率年均提高3个百分点以上。在“护”上出实招。加强重点行业领域环保基础设施建设。加快城镇污水处理及配套收集系统、垃圾回收运储处理设施建设。加强水生态保护，建成重点流域沿岸工业废水及生活污水处理工程。加强耕地和基本农田保护，加大农田改良和修复力度。在“治”上使狠劲。加强大气污染防治，加快清洁能源代替利用。开展矿山地质环境恢复和综合治理。加强农村环境集中连片治理。推进水源地环境整治。到2020年，市中心城区空气质量AQI优良率稳定在85%以上，乡镇千人以上集中式饮用水源地水质达标率达到100%。在“管”上求实效。实行最严格的环境保护制度，落实能源和水资源消耗、建设用地等总量和强度双控行动。用好“两把利剑”，强化“两个问责”，严厉打击污染环境、破坏生态等违法犯罪行为，做到铁腕治污、不欠新账。

　　（七）坚持以“三变”改革为突破，全面推进改革开放创新。着力构建加快发展新体制。深化“三变”改革，完成农村产权确权登记颁证，建立农村产权交易平台和股权交易平台，建立风险基金和农业发展基金，建立股权监管机制、股权退出机制、合同备案机制、法律顾问机制，激发“三变”改革综合效应。深入推进行政审批、国有企业、资源配置、财税金融等体制机制改革，扎实推进教科文卫体等社会事业和司法、社会治理体制改革。完善落实破解民营经济发展难题的政策措施，着力保障民营企业发展需求，到2020年民营经济比重提高到60%。努力开创对外开放新局面。完善对外开放战略布局，抓住国家实施“一带一路”“长江经济带”等战略机遇，积极参与区域分工协作，重点推动与珠三角、长三角、京津冀及东南亚、东盟国家的经贸往来，主动融入黔中经济区、滇中经济区、成渝经济区等区域发展。充分发挥六盘水海关大通关、大开放、大物流作用，把凉都消夏文化节办成开放大平台，不断提高对外开放水平。全力打造创新创业新高地。实施人才引领工程，加强与高校、科研院所产学研合作，推动协同创新合作，实施一批重大科技专项。加强众创、众包、众扶、众筹支撑平台建设，推动新技术、新产业、新业态蓬勃发展。建立创业投资引导基金，支持社会资本参与创业创新。提高社会研发经费占生产总值的比重。加强知识产权保护。

　　（八）坚持以民生改善为根本，着力提高公共服务水平。集中力量实施以脱贫攻坚第一民生工程为重点的“1+7”民生工程，让人民群众有更多的获得感。深入实施教育提升工程。坚持教育优先发展，基本普及15年教育，基本实现县域内义务教育均衡发展。新增省级示范性普通高中5所、省级示范性幼儿园10所。优化职业教育结构，推动高校内涵建设、转型发展，积极发展民族教育、特殊教育、继续教育和民办教育。深入实施健康凉都建设工程。大力实施医疗卫生“十百千”工程，完善市县乡村四级医疗服务网络。积极推动医疗、医保、医药“三医联动”。加快发展“智慧医疗”。加强医疗卫生人才队伍建设。实行最严格的全过程食品安全监管制度。强化计划生育服务管理，落实全面二孩政策。深入实施就业创业促进工程。围绕创建国家级创业型城市目标，大力实施全民创业行动计划，完善创业扶持政策，建立创业服务平台。完善终身职业技能培训制度，健全就业创业服务体系，构建和谐劳动关系。深入实施社会保障兜底工程。推进全民参保登记计划，推进城乡居民基本养老保险制度建设，完善基本医疗保险、大病保险和民政医疗救助“三重医疗保障”，实现城乡居民公平享有医疗保障。全面提升自然灾害综合防范和应对能力。深入实施城乡危旧房改造工程。加强城市老旧住房隐患排查排除，统筹推进城镇保障房、易地扶贫搬迁房建设和农村危房改造，推动城镇保障房向建制镇、工矿区、产业园区和教育园区延伸，努力让人民群众住有所居、住得安全。深入实施公共安全保障工程。筑牢安全生产基础，严格落实安全生产责任制，创新安全生产监督制度，促进安全生产形势彻底好转。深化“平安凉都”建设，完善社会治安综合治理体制机制，推进社会治理精细化，营造平安和谐稳定环境。深入实施文化体育惠民工程。充分挖掘“三线”文化、夜郎文化、民族文化等特色文化资源，加强文化遗产保护利用，打造区域特色文化产业集群。积极发展体育事业和体育产业。统筹发展各项事业，继续实施全民科学素质行动计划，大力支持军队、武警部队和法院、检察院工作，加强国家安全、司法行政和民族、宗教、外事、侨务、对台、科协、人防、档案、红十字、地方志等工作。

　　三、2016年的重点工作

　　今年是“十三五”时期的开局之年，是守底线、走新路、奔小康的关键之年，是去产能、去库存、去杠杆、降成本、补短板的攻坚之年。做好今年经济社会发展各项工作，意义十分重大。

　　今年经济社会发展主要预期目标是：地区生产总值增长12%以上，一产增加值增长7%左右、2000万元以上工业增加值增长10%以上、三产增加值增长13%左右；减少农村贫困人口10万人，实现六枝特区、水城县国家扶贫开发重点县和7个贫困乡镇“减贫摘帽””，130个贫困村退出；公共财政预算收入增长5%左右，公共财政预算支出增长15%左右；500万元以上固定资产投资增长20%以上；社会消费品零售总额增长12%以上；金融机构存款、贷款余额分别增长20%和18%左右；城镇、农村居民人均可支配收入分别增长11%和12%左右；城镇登记失业率控制在4.2%以内；完成省下达的节能减排指标任务。

　　围绕实现全年目标，我们将重点抓好以下工作∶

　　（一）大力推进大扶贫战略行动，确保脱贫攻坚实现良好开局。坚决打好易地扶贫搬迁关键一仗。围绕易地扶贫搬迁“八个精准”，坚持以自然村寨整体搬迁为重点，加强土地和资金需求保障，实施易地扶贫搬迁2.7万人，确保搬得出、稳得住、能致富。统筹推进脱贫攻坚行动。围绕产业脱贫，大力实施特色产业扶贫、乡村旅游扶贫、光伏扶贫、电商扶贫等工程，让贫困户更多分享农业全产业链和价值链增值收益。围绕生态补偿脱贫，加大贫困地区生态保护修复力度，让一批贫困人口就地转成生态保护人员。围绕教育医疗脱贫，落实好贫困家庭学生资助政策，把贫困人口全部纳入重特大疾病救助范围。围绕社会保障兜底脱贫，稳步推进“两线合一、减量提标”，加强最低生活保障与社会救助制度的衔接。强化各类扶贫资源整合。积极争取上级扶贫资金支持。出台扶贫资金和涉农资金整合管理办法，提高资金使用效益。用好对口帮扶、定点帮扶、结对帮扶力量，鼓励社会力量通过多种形式参与扶贫开发。建好用好“扶贫云”和“扶贫专线”。

　　（二）大力推进供给侧结构性改革，加快产业转型升级步伐。全力推进工业转型升级。全面认识去产能、调结构大背景下对我市经济发展的深刻影响，毫不动摇坚定盘活存量与扩大增量的工作定力。准确把握盘活存量是稳增长的重大基础，实施工业“十百千”工程，年内引进4户以上500强企业、实施40户规上企业技术改造、新增1200户以上企业市场主体及80户以上规上企业。推动煤炭产业化危为机，坚持建设提升一批，完成“六化”矿井建设30对，新增机械化矿井10对以上；坚持重组提升一批，支持优强煤业主体兼并重组；坚持转型提升一批，支持黔桂天能等国有和民营大型煤业集团转型发展；坚持融合提升一批，支持煤电联营和“煤电+下游产业”融合发展。煤炭资源就地转化率提高10个百分点以上。积极推进大用户直供电，积极推动发电企业拓展市场，努力提高发电负荷。支持首钢水钢集团转型发展，做强钢铁主业、做优非钢产业。严格执行国家产业政策和能耗、环保、质量、安全等标准，淘汰一批落后产能，清理处置一批“僵尸企业”。深入实施工业“百千万”工程，强化重点行业企业精准服务，为企业转型升级创造条件。准确把握扩大增量是调结构的科学路径，全力发展循环经济，循环经济产值占比提高5个百分点。推进煤炭资源、清洁能源综合利用，完成煤层气抽采量12.37亿立方米、利用率达到42%。推动紫森源煤矸石综合利用项目产业化取得突破。大力发展大数据等新兴产业，引进大数据产业投资资金100亿元、企业100家和人才100名；加快发展农村电子商务，建成4个县级电商运营中心，新建电商服务站（点）150个；全力开发新型建筑材料和装饰材料，加快玄武岩提取高强度纤维及下游产品项目建设，开工一批新型建材产业基地。大力实施提高民营经济比重五年行动计划，全力实施小微型企业“125”计划，支持各类市场主体加快发展，推进大众创业、万众创新。全力推进山地特色农业发展。完成“3155工程”种植100万亩，强化已实施“3155工程”的中后期管护和结构调整优化，提升产业标准和特色产业竞争力。大力推广林菜、林药、林草、林禽等林下种植养殖模式。加快发展特色养殖和山地畜牧业。大力推进国家农业科技园区建设，基本建成水城米箩、六枝郎岱、盘县滑石、钟山大河等11个引领型示范园区，分别新增省级园区、省级重点园区2个以上。引进和培育一批农业经营主体，分别新增省级、市级龙头企业10家和25家以上，新增农民专业合作社100家以上。加快农业现代担保体系建设，扩大农业保险覆盖面。全力推进以山地特色旅游为重点的服务业发展。深入挖掘山地旅游业态，大力实施山地健康旅游目的地城市建设三年行动方案，办好第三届全市旅游文化产业发展大会，推进重点景区提档升级，建成梅花山国际生态休闲度假区。加快培育和引进一批旅游龙头企业，推进旅游地产发展，全力打造“中国凉都”山地特色旅游品牌。实现旅游总收入100亿元，增长35.5%以上。大力发展现代金融业，力争大型金融机构在我市设立区域性互联网金融服务平台，推动浦发银行、光大银行、兴业银行等金融机构在我市设立分支机构，设立六枝村镇银行。加快推进研发设计、商贸会展、家政服务、文化娱乐等服务业发展。

　　（三）大力推进山地特色新型城镇化建设，加快城乡一体化发展。推进农民工市民化。加快户籍制度改革和居住证制度双落地，建立健全“人地钱”三挂钩机制，城镇公租房扩大到非户籍人口。农业转移人口落户城镇4万人以上，全市常住人口城镇化率达到49.5%。提升城市实力。严格执行城市总体规划，大力实施“多规融合”。加快“三地同城化”发展步伐，大力推进城市地下综合管廊等项目建设；开工建设大坪子至董地、乌蒙大道、天湖南路、凤凰东路等道路；启动实施棚户区改造78328户；新建一批停车场、便民超市等公共设施。深入开展“四创”活动，推进城市综合执法，提升城市管理水平。增强城镇活力。启动10个特色小城镇建设，实施“1+N”镇村联动工程21个、“8+X”项目20个。举全市之力承办好第五届全省小城镇建设发展大会，实施六枝特区“整区推进”工程。展现乡村魅力。深入实施六个小康行动计划，全年完成投资17亿元以上，创建点覆盖率增长10%以上，力争达到80%以上。做好传统村落保护工作。加强村寨规划管理，实施农村环境综合整治工程。

　　（四）大力推进投资消费，增强经济增长的拉动力。着力扩大有效投资。完成500万元以上固定资产投资1335亿元以上。强化基础设施投资，完成交通投资100亿元、水利投资60亿元、电力投资6.7亿元。建成沪昆客运专线境内段，加快推进安六城际铁路建设；推进六威高速公路建设，力争建成盘兴高速公路，加快纳兴、六赫高速公路前期工作；推进“四高两快”沿线综合环境建设；启动国省道改造84公里；农村公路行政村通畅率达到100%；加快月照机场盲降系统工程和停机坪扩建工程建设。完成观音岩、阿雨、万营水库主体工程。开工建设中型水库1座以上、小型水库7座；实施2.5万人农村饮水巩固提升工程。全力推进地方直供电输配系统建设，投运35千伏及以上变电站5座、输电线路1336公里。启动实施市中心城区公共免费WIFI项目建设，实现市内4G全覆盖。加大产业投资力度，加强“5个100工程”内容建设，完成工业投资360亿元、房地产开发投资180亿元；优化投资结构，亿元项目投资增长15%以上，产业投资占比保持在45%左右。着力扩大居民消费。围绕扩大住房、汽车、旅游、养老健康、居民用电等消费，落实好刺激、奖励政策。打通保障性住房、易地扶贫搬迁安置住房、农业转移人口落户住房之间的政策性通道，有序化解房地产库存。规划建设一批特色商业街区，开展特色农产品展销会等展会和活动，建设18个标准化菜市场和农贸市场，多措并举促进消费。

　　（五）大力推进生态文明建设，持续改善生态环境质量。加强重点生态工程建设。深入实施“绿色贵州”建设六盘水三年行动计划，完成营造林50万亩、石漠化治理56.52平方公里、水土流失治理48平方公里，森林覆盖率提高3个百分点。加大环境污染治理力度。扎实推进环境污染治理设施建设和大气、水污染防治行动计划，开工建设10个污水处理厂，新建改造污水收集管网60公里。城镇污水处理率、城乡生活垃圾无害化处理率分别达到93%和89%，市中心城区空气质量AQI优良率达到85%以上。强化生态环保监管。全面实行产业准入负面清单，加强环境污染第三方治理。加大环境监管执法力度，严厉打击环境违法行为。

　　（六）大力推进改革开放，有效激发发展动力活力。深化重点领域改革。整市推进“三变”改革，完成确权登记和清产核资，积极开展“两权”抵押贷款试点，推行财政资金股权化改革，创造条件建立产权交易中心。深化商事制度改革，加快国有企业分类改革，推进供销合作社综合改革。深化财税体制改革，完善预算绩效管理机制。实施机关事业单位养老保险制度改革。全力扩大对外开放。加快六盘水海关建设，力争9月底前建成开关。用好酒博会、茶博会等重大活动平台。加强与国内外和周边地区区域经济合作。深化大连对口帮扶工作。围绕特色优势产业，大力开展精准招商，完成省内外招商引资项目实际到位资金1000亿元。加大人才培养引进力度，每万人人才资源数达到980人。

　　（七）大力推进民生改善，切实维护社会和谐稳定。抓好就业根本民生。大力实施就业创业促进工程，引导支持高校毕业生、返乡农民工、复退军人、妇女创业就业，帮助就业困难群体和特殊群体就业，城镇新增就业9.37万人以上。抓好教育长远民生。六枝特区、盘县、钟山区基本普及15年教育和盘县县域内义务教育基本均衡通过省级验收。实现农村义务教育营养改善计划全覆盖。建成高中教育城。新增省级示范性普通高中1所以上。推动六盘水师范学院向应用型本科院校转型，力争六盘水职业技术学院成功创建省级示范职业技术学院。加强乡村教师队伍建设。抓好医疗健康基本民生。实现乡镇卫生院标准化建设全覆盖、县级以上公立医院远程医疗全覆盖。加快推进分级诊疗和优质医疗资源下沉。加强重大疾病和地方病防控。扎实抓好国家卫生城市创建工作。坚持计划生育基本国策，强化服务和管理，落实全面二孩政策。抓好社会保障普惠民生。巩固和扩大社会保障覆盖范围，提高城乡居民最低生活保障和新农合政府补助标准。统筹推进城乡社会救助体系建设，落实农村“三留守”人员关爱救助保障政策。抓好安全稳定重要民生。深入推进“2221”安全生产体系建设，加强应急应对能力建设，实施社会治安防控体系建设工程，做好信访维稳工作，严防各类重特大事故事件发生，切实维护社会和谐稳定。

　　扎实办好“十件民生实事”。（1）压缩6%的行政经费用于教育精准扶贫，对2万建档立卡农村贫困家庭学生上高中、大学免除（补助）学杂费。（2）扶持微型企业1300户，带动就业6500人。公益性岗位安置就业困难人员6500人以内。（3）高中教育城实现招生。建设28所农村幼儿园、32个标准化农村寄宿制学校和80个农村留守儿童之家。（4）基本建成凉都医院。所有农村中小学配置校医。提高城镇居民基本医疗保险和新农合补助标准。（5）农村、城市低保标准分别提高18%和10%，提高残疾人补助标准。（6）基本建成水城河综合治理工程。完成农村危房改造1万户。（7）新建、改建农村通组路和串户路796公里。新增公共停车位1280个。（8）县级以上城市建成区、高速公路和高速铁路沿线移动网络有效信号实现全覆盖。（9）完成5个传统村落消防安全改造。（10）实施26470户“三改三治”及庭院硬化、21个乡镇垃圾收集处理、5个行政村集中式饮用水源地保护，推进166个行政村便民服务设施、161个行政村文体活动广场及设施建设、234个自然村寨污水治理、5203盏照明设施安装。新建1个县级老年体育活动中心，新建和改造30个全民健身路径工程，新建50个村级农民体育健身工程。

　　四、加强政府自身建设

　　各位代表！完成“十三五”规划目标任务，全面建成小康社会，对政府工作提出了新的更高要求。我们将加快建设法治政府、创新政府、廉洁政府和服务型政府，不断增强执行力和公信力。

　　（一）围绕执政有章，加强法治建设。坚持把政府工作全面纳入法治轨道，所有行政行为都要于法有据，任何部门都不得法外设权。出台《市人民政府规章程序办法》。完善权力清单和责任清单，公开权力运行流程和审批流程。完善和深化重大决策合法性审查机制，建立政府决策执行纠偏机制。自觉接受人大法律和工作监督、政协民主监督、新闻舆论和社会公众监督，高度重视民主党派、工商联和无党派人士的意见建议，充分发挥工青妇等人民团体的桥梁和纽带作用。政府所有工作都要全面接受人民监督，充分体现人民意愿。

　　（二）围绕从严从实，加强作风建设。认真践行“三严三实”，自觉向中央、省委和市委看齐，坚决维护中央、省委和市委权威。坚持把纪律建设摆在更加突出位置，坚决惩处失职渎职、懒政怠政。以行政权力瘦身为市场主体强身，推动招商引资代办中心、中央和省属单位公共服务事项进驻政务服务中心，最大限度精简办事程序，简化办事环节，缩短办理时限。切实改进服务方式，实现市县乡政府行政事务“一张网”，畅通群众办事“最后一公里”，让“信息多跑路”“群众少跑腿”，变“群众来回跑”为“部门协同办”。

　　（三）围绕担当作为，加强能力建设。坚持用五大发展理念武装头脑、指导实践、推动工作，认真学习研究国家政策，吃透上情、把握市情、了解外情，找准我市落地落实的切入点和结合点，推动弯道取直、后发赶超。始终把干事创业谋发展、狠抓落实惠民生作为最大责任，以“功成不必在我”“功夫必须在我”的境界，善于“扣扣子”、勇于“担担子”、勤于“钉钉子”，敢作敢为、善作善成，以政府的效能指数提升全市的发展指数。强化正向激励，严肃行政问责，让“实干家”有为有位，让“太平官”无处可混。

　　（四）围绕从严治政，加强廉政建设。全面落实党风廉政建设主体责任，自觉遵守廉洁自律准则和纪律处分条例，充分运用大数据提升政府治理能力，切实管好公共权力、公共资金、公共资源、公务人员，扎紧“制度铁笼”，打造“数据铁笼”，让权力运行处处留痕，在政府系统营造不敢腐、不能腐、不想腐的良好氛围。加强行政监察和审计监督，深化拓展民生监督工作，以零容忍的态度惩治腐败，努力实现干部清正、政府清廉、政治清明。

　　各位代表！回顾“十二五”，我们硕果累累；展望“十三五”，我们信心满满！让我们紧密团结在以习近平同志为总书记的党中央周围，在省委、省政府和市委的坚强领导下，团结带领全市各族干部群众，鼓起劲来抓发展，弯下腰来拔穷根，携起手来奔小康，为夺取全面建成小康社会伟大胜利而努力奋斗！
